# Supplementary material for: Incidence, Compliance, and Risk Factor Associated with Central Line-Associated Bloodstream Infection (CLABSI) in Intensive Care Unit (ICU) Patients: A Multicenter Study in an Upper Middle-Income Country
Source: Antibiotics (Basel). 2025 Mar 7;14(3):271. doi: 10.3390/antibiotics14030271 (PMC11939773; doi:10.3390/antibiotics14030271)
Supplement: Supplementary file 1 [file antibiotics-14-00271-s001.zip › antibiotics-3477605-supplementary-File S1.pdf]

Guideline of Data Collection

Central Line-Associated Bloodstream Infection (CLABSI)

Trans Research Grant Scheme – Evaluation and Recommendation of Infection Control in Malaysia (TRGS- MYERIC)

2024

Instruction for Completion of CLABSI data surveillance Form

No	Data Field	Instruction for data entry
		**FO1: DEMOGRAPHIC**
**1**	Record ID	Record ID number will be auto entered by the system
**2**	Name of the investigator	Required. Enter the name of personnel who key the data in CRF
**3**	Date of survey	Required. Record the date of the survey entered using this format: DD/MM/YYYY
**4**	Year of survey	Required. Specify which year the survey collected. Check 2022, 2023 and 2024
**5**	Month of the survey	Required. Check the correct month of the survey data collected.
**6**	Patient CRF CLABSI ID	Each hospital with its own unique ID (e.g.) (CLABSI/XXX/2024/Apr 0001) for XXX
**7**	Name of patient	Required. Enter the full name of the patient.<br>Identifier.
**8**	Registration number (RN) of patient	Required. Enter the numeric patient RN number.<br>Please add ‘0’ in front if the RN number is not 8 digit eg. 568425 = 00568425
**9**	Age of patient	Age will be auto entered by the system.
**10**	Birth date of patient	Required. Record the date of the patient birth using this format: DD/MM/YYYY
**11**	Gender	Required. Check Male, Female, or Other to indicate the gender of the patient
**12**	Ethnicity of patient	Required. Specify if the patient is either Malay, Chinese, Indian or Others. If Other, please state ethnicity.
**13**	Weight of patient (kg)	Required. Enter the weight of patient in this format: kilogram (kg)
**14**	Height of patient (m)	Required. Enter the height of patient in this format: metre (m)

**15**	BMI of patient	BMI will be auto calculated by the system.
	**ADMISSION DETAILS**	
**16**	Admission date in hospital	Enter the date of patient admitted in hospital or emergency ward using this format: DD/MM/YYYY
**17**	Discharge date in hospital	Enter the date of patient discharged from hospital ward using this format: DD/MM/YYYY
**18**	Duration of stay in hospital	Duration of stay will be auto calculated by system.
**19**	Admission date in ICU	Required. Enter the date of patient admitted in Intensive Care Unit (ICU) using this format: DD/MM/YYYY
**20**	Discharge date in ICU	Required. Enter the date of patient discharged from the Intensive Care Unit (ICU) using this format: DD/MM/YYYY
**21**	Duration of stay in ICU	Duration of stay will be auto calculated by system.
**22**	More than 48hr admission in ICU	Specify Yes or No. If patient were admitted more than 48hr starting from admission in the ICU.<br>* This is to check for site of acquisition
**23**	Clinical diagnosis of patient in ICU	State the clinical diagnosis of patient during ICU stay.<br>1. Gastrointestinal bleeding<br>2, Trauma<br>3, Immunological disease<br>4, Disorder of consciousness<br>5, Organ transplantation<br>6, Multiple organ failure<br>7, Shock<br>8, Pancreatitis<br>9, acute respiratory distress syndrome (ARDS)<br>10, Sepsis<br>11, Pneumoniae<br>12, Intrabdominal infection (IAD)<br>13, Others, please specify……………………

MEDICAL DEVICES AND PROCEDURES		
**24**	Dialysis within 30 days	Specify Yes or No if patient undergo dialysis within 30 days.
**25**	Surgery within 30 days	Specify Yes or No if patient undergo dialysis within 30 days.
**26**	Ambulatory oncology therapy within 30 days	Specify Yes or No if patient undergo oncology therapy within 30 days.
**27**	Medical devices presented during ICU stay	○ Peripheral IV catheter<br>Specify Yes or No if there is presence of peripheral venous line. Peripheral intravenous (IV) lines, catheters or cannulas are indwelling single-lumen plastic conduits that allow fluids, medications and other therapies such as blood products to be introduced directly into a peripheral vein of forearm or hand.
		○ Arterial line<br>Specify Yes or No if there is presence of arterial lines. Arterial catheterization is a procedure that is common to the intensive care and the operating room settings. It involves placement of a catheter into the lumen of an artery to provide at minimum a continuous display blood pressure with access to frequent arterial blood sampling.
		○ Urinary Catheter
		○ Mechanical Ventilation<br>Specify Yes or No if mechanical ventilation was performed to patient within that time. A mechanical ventilator is a machine that helps a patient breathe (ventilate) when they are having surgery or cannot breathe on their own due to a critical illness. The patient is connected to the ventilator with a hollow tube (artificial airway) that goes in their mouth and down into their main airway or trachea. They remain on the ventilator until they improve enough to

		breathe on their own.<br>○ None
**28**	Co-morbidities	Required. Check box (can be more than 1)<br>Check all the co-morbidities:<br><br>○ DM<br>○ Hypertension<br>○ Obesity<br>○ Cardiovascular disease<br>○ Atrial fibrillation<br>○ Venous thromboembolism<br>○ CKD/ESRF<br>○ CVA/stroke<br>○ Chronic liver disease<br>○ Chronic pulmonary disease<br>○ Connective tissue disease<br>○ Tumor without metastases<br>○ Cystic fibrosis<br>○ Dementia or other neurological condition<br>○ Dyslipidemia<br>○ Immunocompromised<br>○ Others, State other co-morbidity if not listed……..<br>○ None
**29**	If Immunocompromised	If patient is a immunosuppressed means having a weekend immune system due to following item listed. Check box the appropriate. If there is other immunosuppressed, please state.<br>Some people who are immunocompromised (have a weakened immune system) are more likely to get sick with COVID-19 or be sick for a longer period. People are considered to be moderately or severely

		immunocompromised due to several types of conditions and treatments, including:<br>▪ Active treatment for solid tumor and hematologic<br>▪ Malignancies<br>▪ Receipt of solid-organ transplant and taking immunosuppressive therapy<br>▪ Receipt of chimeric antigen receptor (CAR)-T-cell therapy or hematopoietic cell transplant (HCT) (within 2 years of transplantation or taking immunosuppressive therapy).<br>▪ Moderate or severe primary immunodeficiency (e.g., DiGeorge syndrome, Wiskott-Aldrich syndrome) Advanced or untreated HIV infection (people with HIV and CD4 cell counts less than 200/mm3, history of an AIDS-defining illness without immune reconstitution, or clinical manifestations of symptomatic HIV)<br>▪ Active treatment with high-dose corticosteroids (i.e., 20 or more mg of prednisone or equivalent per day when administered for 2 or more weeks), alkylating agents, antimetabolites, transplant-related immunosuppressive drugs, cancer chemotherapeutic agents classified as severely immunosuppressive, tumor necrosis factor (TNF) blockers, and other biologic agents that are immunosuppressive or immunomodulatory.
**30**	Charlson Comorbidity Index	The Charlson Comorbidity Index is a method of categorizing comorbidities of patients based on the International Classification of Disease (ICD). Please use this link to calculate CCI: https://www.medicalalgorithms.com/comorbidity-index-charlson

**31**	APACHE II Score	The Acute Physiology and Chronic Health Evaluation (APACHE) II was proposed by Knauss WA, Draper EA, et all as a severity of disease classification system in 1985 scoring system. It was used to evaluate the disease severity during the hospitalization in the ICU. The score is calculated within 24 hours of admission of a patient to an intensive care unit (ICU) and in this scoring score from 0 to 71 is computed based on several measurements. https://clincalc.com/IcuMortality/APACHEII.aspx

No	Data Field	Instruction for data collection

FO2: CATHETER DETAILS & COMPLIANCE

[can add instance if patient have more than one central line at same admission in ICU, For example, '1' or '01' represents catheter one, '2' or '02' represents catheter two, and so on, in accordance with the catheter's placement order. Click 'add instance' for each catheter.]

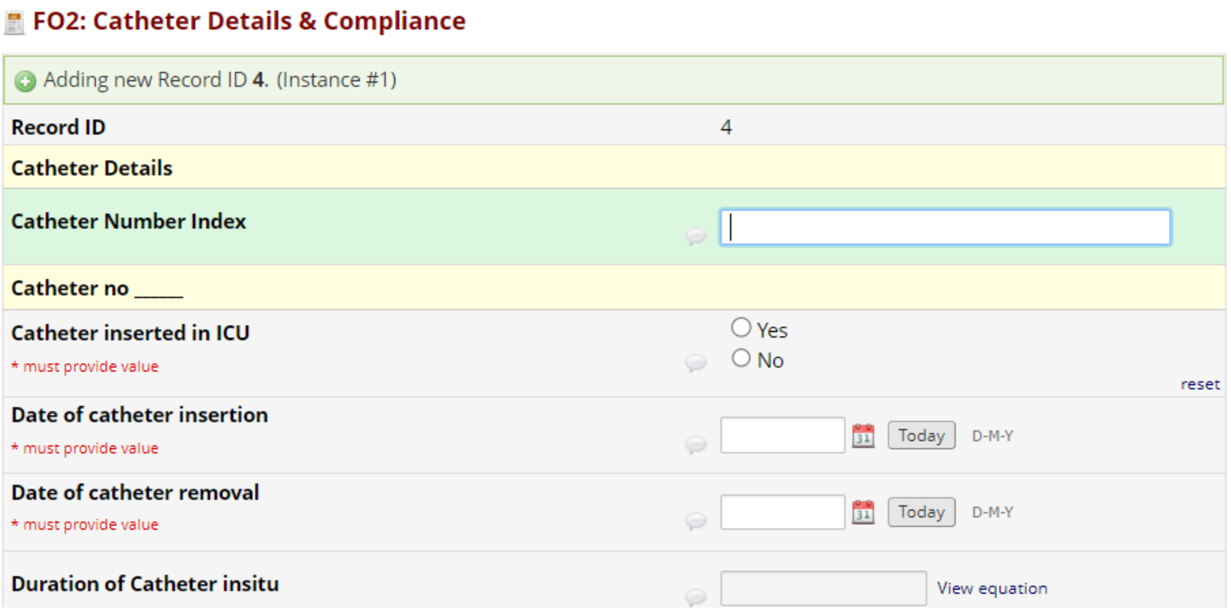

	Catheter Number Index	Please indicate catheter no (**If a patient has more than one central line, please record each catheter with a catheter index number. This index number is crucial for accurate documentation and will be used when keying in the daily review form for the**

		**respective catheter. For example, '1' or '01' represents catheter one, '2' or '02' represents catheter two, and so on, in accordance with the catheter's placement order. Click 'add instance' for each catheter.**
	Catheter number	Will automatically index the catheter number index
	Catheter inserted in ICU	Specify Yes or No if the catheter was inserted in ICU
	Date of catheter insertion	Please state the date of catheter<br>insertion using this format: DD/MM/YYYY<br>(If catheter inserted, outside ICU and unable to retrieve the insertion details, please leave it blank)
	Date of catheter removal	Enter the date of catheter removal using this format: DD/MM/YYYY
	Duration of catheter insitu	Duration of catheter insitu will be auto calculated by the system.
**Type of central line**		☐ Non tunnelled<br>☐ tunnelled<br>☐ totally implantable<br>Tunneled catheters are implanted surgically (by creating a subcutaneous track before entering vein) into the jugular, subclavian or femoral vein for long term (weeks to months) use such as chemotherapy or hemodialysis. Non- tunneled catheters are temporary central venous catheter that are inserted percutaneously. Totally implantable venous access devices, which are placed entirely under the skin tissue (no skin exit site)
	Type of central line	Please identify if patient have :<br><br>○ Non tunneled<br>○ Tunneled<br>○ Totally Implantable<br><br>Central line (CL): An intravascular catheter that terminates at or close to the heart, or in one of the great vessels AND is used for infusion, withdrawal of blood, or hemodynamic monitoring.<br><br>I. Permanent central line includes:

		a. Tunneled catheters, including tunneled dialysis catheters.<br>b. Implanted catheters (including ports)<br><br>○ Tunneled catheters are implanted surgically (by creating a subcutaneous track before entering vein) into the jugular, subclavian or femoral vein for long term (weeks to months) use such as chemotherapy or hemodialysis.<br>○ Temporary central line: A non-tunneled, non-implanted catheter. Non- tunneled catheters are temporary central venous catheter that are inserted percutaneously.<br>○ Totally implantable venous access devices, which are placed entirely under the skin tissue (no skin exit site)
	Site of catheter insertion tunneled	If patient have tunneled, please identify the site of catheter insertion:<br>i. Femoral<br>ii. Jugular<br>iii. Subclavian vein<br>iv. Others, please state..
	Number of lumens for tunneled	Specify the number of lumens for tunneled catheter:<br>i. Single<br>ii. Double<br>iii. Triple/more
	Use of lock solution for tunneled	Specify the number of lock solution:<br>i. Anticoagulant<br>ii. Citrate<br>iii. Antibiotic<br>iv. No lock solution.

		Locking is defined as the injection of a limited volume of liquid following the catheter flush, for the period of time when the catheter is not in used to prevent intraluminal clot formation and/or catheter colonization.
	Site of catheter insertion non- tunneled	If patient have non- tunneled, please identify the site of catheter insertion:<br>i. Femoral<br>ii. Jugular<br>iii. Subclavian vein<br>iv. Others, please state..
	Number of lumens for non-tunneled	Specify the number of lumens for non- tunneled catheter:<br>i. Single<br>ii. Double<br>iii. Triple/more
	Use of lock solution for non- tunneled	Specify the number of lock solution:<br>i. Anticoagulant<br>ii. Citrate<br>iii. Antibiotic<br>iv. No lock solution.<br><br>Locking is defined as the injection of a limited volume of liquid following the catheter flush, for the period of time when the catheter is not in used to prevent intraluminal clot formation and/or catheter colonization.
	Site of catheter insertion totally implantable	If patient have totally implantable, please identify the site of catheter insertion:<br>i. Femoral<br>ii. Jugular<br>iii. Subclavian vein<br>iv. Others, please state..
	Number of lumens for totally implantable	Specify the number of lumens for totally implantable catheter:<br>i. Single

		ii. Double<br>iii. Triple/more
	Use of lock solution for totally implantable	Specify the number of lock solution:<br>i. Anticoagulant<br>ii. Citrate<br>iii. Antibiotic<br>iv. No lock solution.<br><br>Locking is defined as the injection of a limited volume of liquid following the catheter flush, for the period of time when the catheter is not in used to prevent intraluminal clot formation and/or catheter colonization.
**INSERTION DETAILS & COMPLIANCE TO BUNDLE**		

**Insertion Details and Compliance to bundle** _____		
**Category of catheter insertion**	○ Elective<br>○ Emergency<br>○ Not Documented	reset
**Category of operator**	○ HO<br>○ MO<br>○ Specialist<br>○ Not Documented	reset
**Ultrasound guided catheter insertion**	○ Yes<br>○ No<br>○ Not Documented	reset
**Hand Hygiene**	○ Yes<br>○ No<br>○ Not Documented	reset
**Maximal barrier precaution**	○ Yes<br>○ No<br>○ Not Documented	reset
**Chlorhexidine antisepsis**	○ Yes<br>○ No<br>○ Not Documented	reset

	Category of catheter insertion	Specify if the insertion was elective or emergency or not documented.<br>Elective surgery means that the surgery can be scheduled in advance. Emergency surgery meaning surgery that is done

		because of an urgent medical condition (may even be life threatening). Click non-documented if the catheter insertion checklist is empty or missing or untraceable.
	Category of operator	Specify who performs the insertion: specialist, medical officer, or houseman.
	Ultrasound guided catheter insertion	Specify Yes or No or Not documented if the operator uses the ultrasound prior to catheter insertion. Click non-documented if the catheter insertion checklist is empty or missing or untraceable.
	Hand Hygiene	Specify Yes or No or Not documented if the operator performs the hand hygiene. Hand hygiene is a way of cleaning one's hands that substantially reduces potential pathogens (harmful microorganisms) on the hands. Hand hygiene is considered a primary measure for reducing the risk of transmitting infection among patients and health care<br>personnel. The 5 moments of hand hygiene is before touching a patient, before clean/aseptic procedures, after body fluid exposure/risk, after touching a patient and after touching patient surroundings. Click non-documented if the catheter insertion checklist is empty or missing or untraceable.
	Maximal barrier precaution	Specify Yes or No or Not documented.<br>The maximal barrier precaution requires that the person inserting the CVC wear a head cap, face mask, sterile body gown, sterile gloves and use a full-size sterile drape around the insertion site.
	Chlorohexidine antiseptic	Specify Yes or No or Not documented if the chlorhexidine antiseptic was used. The chlorohexidine is an antiseptic and disinfectant is effective against wide range of both gram positive and gram-negative bacteria.

FO3: DAILY REVIEW

(* Must enter the daily review for catheter in situ in ICU)

**Catheter number**	
**Catheter number of ____**	
**Date of review**	Today D-M-Y
**Ongoing long term intravenous therapy**	○ Yes<br>○ No<br>○ Not documented<br>reset
**On Inotropes in the last 24hr**	○ Yes<br>○ No<br>○ Not Documented<br>reset
**On TPN in the last 24hr**	○ Yes<br>○ No<br>○ Not Documented<br>reset<br>Total Parenteral Nutrition
**On Dialysis**	○ Yes<br>○ No<br>○ Not Documented<br>reset
**Unable to set peripheral venous lines**	○ Yes<br>○ No<br>○ Not Documented<br>reset
**Need for CVP monitoring**	○ Yes<br>○ No<br>○ Not Documented<br>reset
**Need for infusion of hypertonic solution**	○ Yes<br>○ No<br>○ Not Documented<br>reset

**No**	**Data Field**	**Instruction for data collection**
	Catheter Number	Please indicate catheter no according to the catheter number inserted in the FO2. (**For example, '1' or '01' represents catheter one, '2' or '02' represents catheter two, and so on, in accordance with the catheter's placement order. Click 'add instance' for each daily review according to catheter insitu.**
	Date of Review	Enter the date of review performed using this format: DD/MM/YYYY
	On going long terms intravenous therapy	Specify Yes or No or Not documented if patient was on long term intravenous therapy.
	On Inotropes in the last	Specify Yes or No or Not documented if patient was on inotropes.

	24hr	Inotropes are drugs that change the force of the heart's contractions.
	On TPN in the last 24 hours	Specify Yes or No or Not documented is patient under total parental nutrition. Total parenteral nutrition (TPN) supplies all daily nutritional requirements. TPN can be used in the hospital or at home. Because TPN solutions are concentrated and can cause thrombosis of peripheral veins, a central venous catheter is usually required. TPN may be the only feasible option for patients who do not have a functioning gastrointestinal (GI) tract or who have disorders requiring complete bowel rest, such as the following:<br>▪ Some stages of ulcerative colitis<br>▪ Bowel obstruction<br>▪ Certain pediatric GI disorders (eg, congenital GI anomalies, prolonged diarrhea regardless of its cause)<br>▪ Short bowel syndrome due to surgery
	On Dialysis	Specify Yes or No or Not documented if patient on dialysis. Dialysis is a procedure to remove waste products and excess fluid from the blood when the kidney stop working properly. Hemodialysis patients are at a high risk for infection because the process of hemodialysis requires frequent use of catheters or insertion of needles to access the bloodstream.
	Unable to set peripheral venous lines	Specify Yes or No or Not documented. A peripheral venous catheter is a thin, flexible tube that is inserted into a vein. It is usually inserted into the lower part of the arm or the back of the hand. It is used to give intravenous fluids, blood transfusions, chemotherapy, and other drugs.
	Need for CVP monitoring	Specify Yes or No or Not documented. Central venous pressure (CVP), an estimate of right atrial pressure, has been used to assess cardiac preload and volume status in critically ill patients, assist in the diagnosis of right-sided heart failure, and guide fluid resuscitation. Central venous pressure (CVP) monitoring is used

		to assess the fluid status of patients in critical care settings

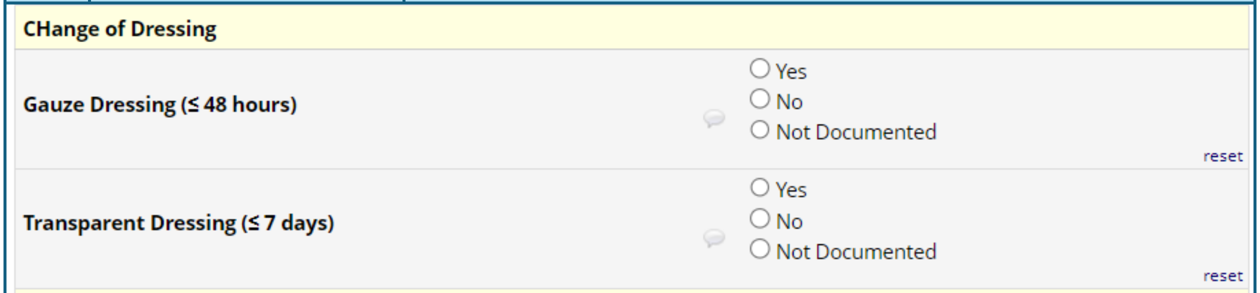

CHange of Dressing

Gauze Dressing (≤ 48 hours)	○ Yes ○ No ○ Not Documented (reset)
Transparent Dressing (≤ 7 days)	○ Yes ○ No ○ Not Documented (reset)

No	Data Field	Instruction for data collection
	Gauze Dressing (≤ 48 hours)	Specify Yes or No or Not documented. Gauze will be used if exit site is not visible 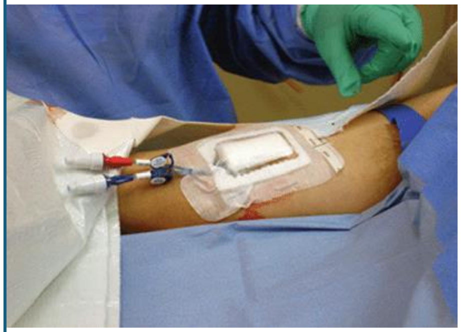
	Transparent Dressing (≤ 7 days)	Specify Yes or No or Not documented. Transparent dressing will be used when exit site is more visible 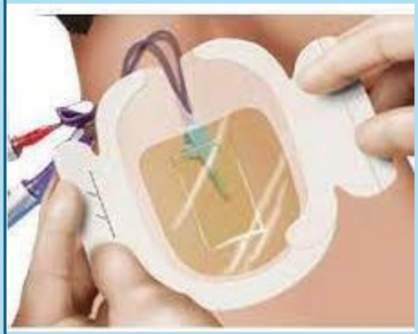

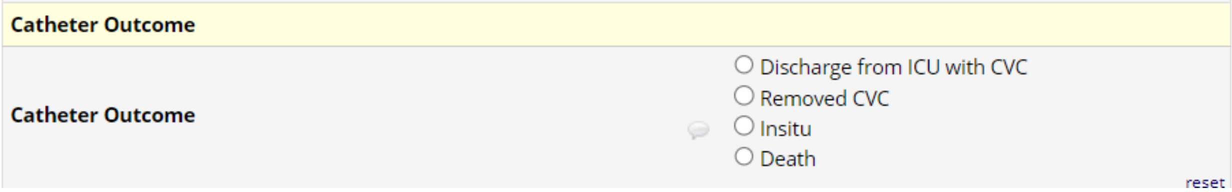

Catheter Outcome

Catheter Outcome	○ Discharge from ICU with CVC ○ Removed CVC ○ Insitu ○ Death (reset)

No	Data Field	Instruction for data collection
	Catheter Outcome	Specify if the catheter is in place/ insitu or removed or discharge from ICU with CVC in place or death.

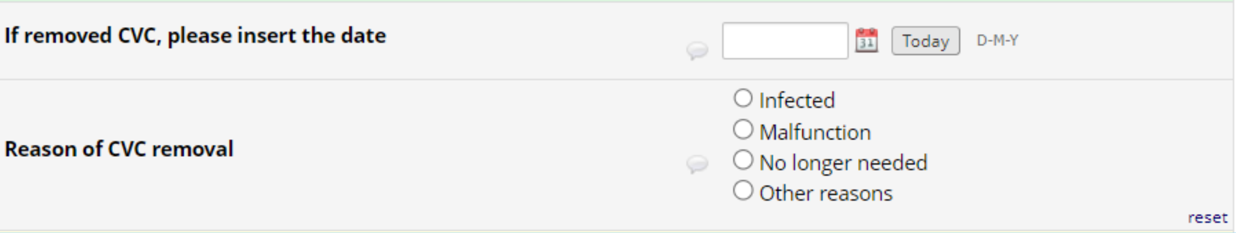

If removed CVC, please insert the date — Today D-M-Y

Reason of CVC removal
- Infected
- Malfunction
- No longer needed
- Other reasons

reset

No	Data Field	Instruction for data collection
	IF removed CVC, please insert the date	Enter the date of removal using this format: DD/MM/YYYY
	Reason of CVC removal	Specify if the CVC is removed because of infected, malfunction or no longer needed or other reasons................

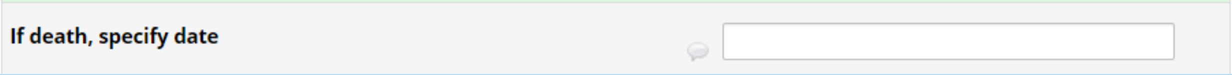

If death, specify date

	If death	Enter the date of death with catheter using this format: DD/MM/YYYY

FO4 : MICROBIOLOGY CULTURE

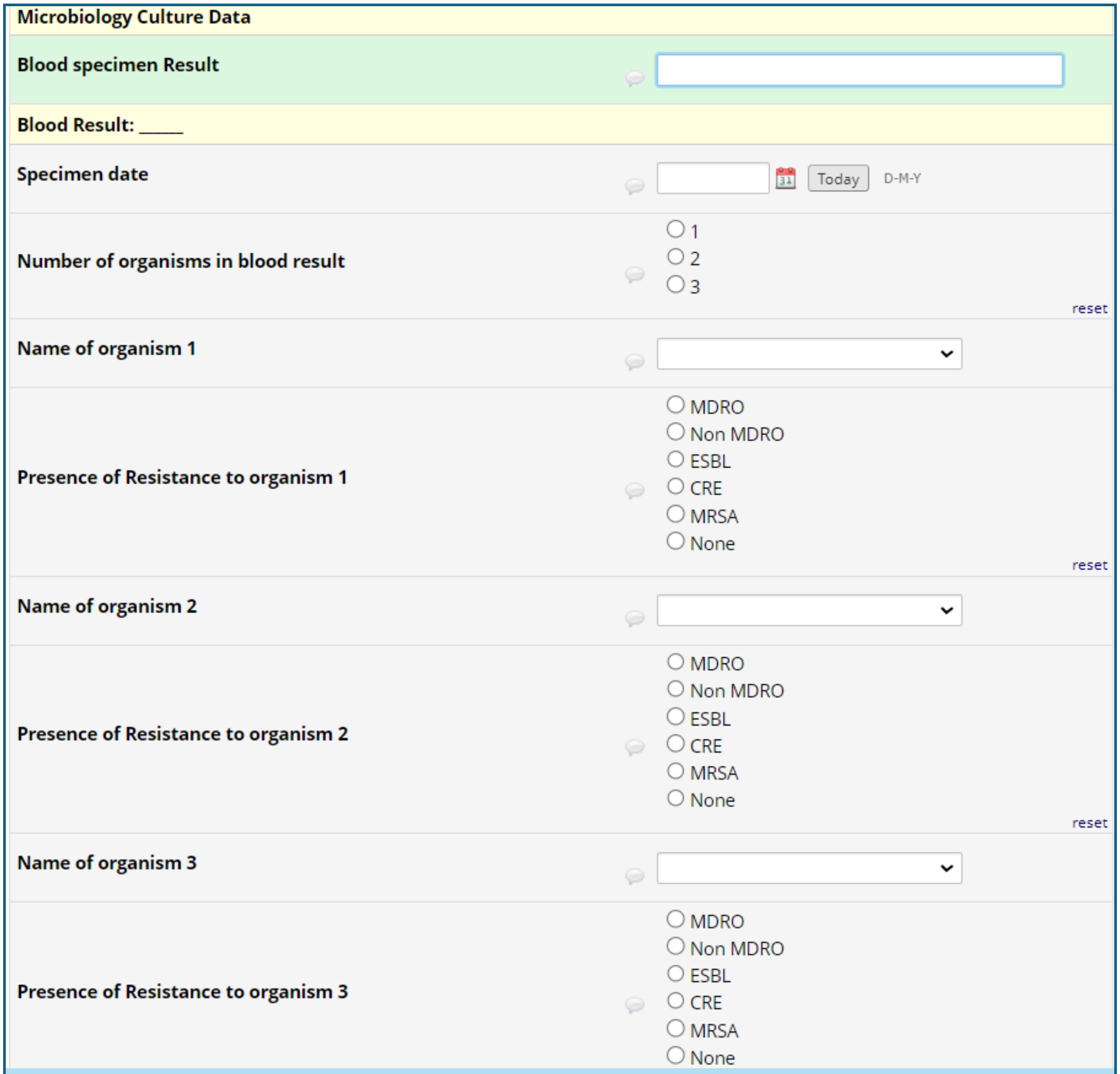
Microbiology Culture Data

Blood specimen Result

Blood Result: _____

Specimen date Today D-M-Y

Number of organisms in blood result
- 1
- 2
- 3

reset

Name of organism 1

Presence of Resistance to organism 1
- MDRO
- Non MDRO
- ESBL
- CRE
- MRSA
- None

reset

Name of organism 2

Presence of Resistance to organism 2
- MDRO
- Non MDRO
- ESBL
- CRE
- MRSA
- None

reset

Name of organism 3

Presence of Resistance to organism 3
- MDRO
- Non MDRO
- ESBL
- CRE
- MRSA
- None

No	Data Field	Instruction for data collection
	Blood specimen result	Add in all the blood result taken during patient stay in ICU with catheter in place.<br>Write each blood result as “1”, next blood result as “2” and so on. Add instance to add new blood result.
	Specimen date	Date of collection for the blood specimen in the ICU using this format DD/MM/YYYY
	Number of organisms in blood result	Identify 1,2 or 3 number of organism found in the dated blood result.
	Name of organism	Required. Select ONE organism from the list OR click

		others to specify the organism name. Please write full organism name.<br>Click on the link to see all the listed organism's name. master-organism-com-commensals-lists.xlsx (live.com)<br>Up to three organisms may be reported. If multiple organisms are identified enter the organisms judged to be the most important cause of infection as #spp1, the next most as #spp2 and the least as #spp3 (usually this order will be indicated on the laboratory report). If the species is not given on the lab report or is not found on the NHSN drop down list, then enter on other organisms spp column.
	Presence of resistance	Required. Click on the resistance mode either MDRO, Non MDRO, ESBL, CRE or MRSA.<br>Multidrug resistance (MDR) was defined as isolates resistant to at least three drugs in the following classes: β-lactams, carbapenems, aminoglycosides, and fluoroquinolones. Extensive drug resistance (XDR) was defined as nonsusceptibility to at least one agent in all but two or fewer antimicrobial categories (i.e. bacterial isolates remain susceptible to only one or two categories). MRSA, MSSA, VRE, CephR-Klebsiella, CRE (E.coli, Klebsiella pneumoniae, Klebsiella oxytoca, Klebsiella aerogenes, or Enterobacter), MDR-Acinetobacter or C.difficile.

Infection Details

Blood Result: _____

State the infection or colonization origin

- ○ HAI
- ○ HAC
- ○ HCAI
- ○ HCAC
- ○ CAI
- ○ CAC

reset

The three types of infections or colonization will be considered: Hospital acquired infection (HAI)is defined as isolation of organism from a sterile site culture >48 h after admission and if there were no signs or symptoms of infection at admission OR - SSI criteria met (surgery in previous 30 days/ with implant 1year), OR - Discharged from acute care hospital < 48 hours ago, OR - CDI and discharged from acute care hospital < 28 days ago OR Community acquired infection (CAI) is defined as isolation of organism from a sterile site <48 h of admission and if the episode did not fit the above conditions. Health care associated infection (HCAI) is defined as infection that occurred amongst those that had contact with the healthcare system within the previous 3 months; or It also includes those who came from the nursing home or on hemodialysis.

Was BSI primary or secondary?

- ○ Primary
- ○ Secondary

reset

A Laboratory Confirmed Bloodstream Infection (LCBI) that is not secondary to an infection at another body site.Secondary BSI: A BSI that is thought to be seeded from a site-specific infection at another body site Reference: (CDC -Bloodstream Infection Event (Central Line-Associated Bloodstream Infection and Non-central Line Associated Bloodstream Infection (January 2022) : page 4-3)

No	Data Field	Instruction for data collection
	State the infection or colonization origin	Required. Check box the type of infections or colonization<br>The three types of infections or colonization will be considered:<br>○ HAI: Hospital acquired infection<br>○ HAC: Hospital acquired colonization<br>○ HCAI: Healthcare associated infection<br>○ HCAC: Healthcare associated colonization<br>○ CAI: Community acquired infection<br>○ CAC: Community acquired colonization<br>Hospital acquired infection (HAI)is defined as isolation of organism from a sterile site culture >48 h after admission and if there were no signs or symptoms of infection at admission OR<br>▪ SSI criteria met (surgery in previous 30 days/ with implant 1year), OR<br>▪ Discharged from acute care hospital < 48 hours

		ago, OR<br>▪ CDI and discharged from acute care hospital < 28 days ago OR<br>Community acquired infection (CAI) is defined as isolation of organism from a sterile site <48 h of admission and if the episode did not fit the above conditions.<br><br>Health care associated infection (HCAI) is defined as infection that occurred amongst those that had contact with the healthcare system within the previous 3 months; or It also includes those who came from the nursing home or on hemodialysis.
	Was BSI primary or secondary	Specify if patient is primary or secondary BSI.<br><br>The types will be considered:<br><br>Primary bloodstream infection (BSI): A Laboratory Confirmed Bloodstream Infection (LCBI) that is not secondary to an infection at another body site. The primary BSI – CLABSI Central line-associated BSI (CLABSI): A laboratory confirmed bloodstream infection where an eligible BSI organism is identified, and an eligible central line is present on the LCBI DOE or the day before. (Reference: (CDC -Bloodstream Infection Event (Central Line-Associated Bloodstream Infection and Non-central Line Associated Bloodstream Infection (January 2022) : page 4-5)<br><br>Secondary BSI: A BSI that is thought to be seeded from a site-specific infection at another body site (see Appendix B. Secondary BSI Guide and CDC/NHSN Surveillance

		Definitions for Specific Types of Infection [Ch-17], UTI [Ch-7], Pneumonia (Ch-6), and SSI (Ch-9)

FO5: TREATMENT REGIMEN

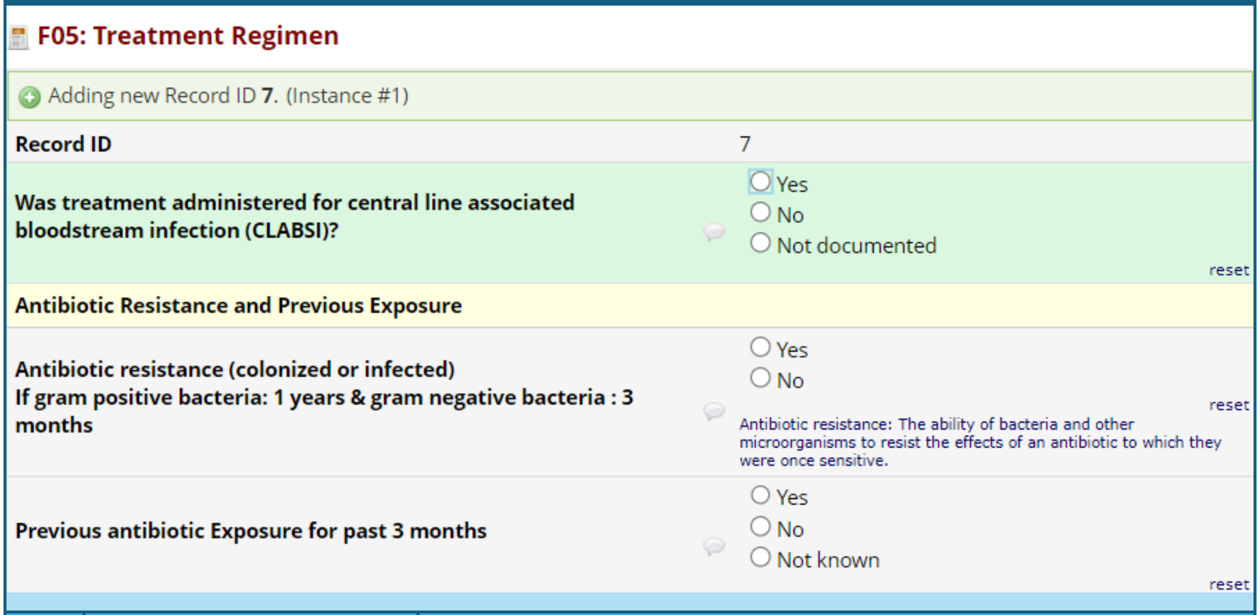
F05: Treatment Regimen

Adding new Record ID 7. (Instance #1)

**Record ID**	7
**Was treatment administered for central line associated bloodstream infection (CLABSI)?**	○ Yes<br>○ No<br>○ Not documented<br>reset
**Antibiotic Resistance and Previous Exposure**	
**Antibiotic resistance (colonized or infected)**<br>**If gram positive bacteria: 1 years & gram negative bacteria : 3 months**	○ Yes<br>○ No<br>reset<br>Antibiotic resistance: The ability of bacteria and other microorganisms to resist the effects of an antibiotic to which they were once sensitive.
**Previous antibiotic Exposure for past 3 months**	○ Yes<br>○ No<br>○ Not known<br>reset

No	Data Field	Instruction for data collection
	Was treatment administered for central line associated bloodstream infection (CLABSI)	Required. Specify Yes or No or Not documented. If patient was positive BSI and also CLABSI, please check treatment was given to resolve CLABSI.

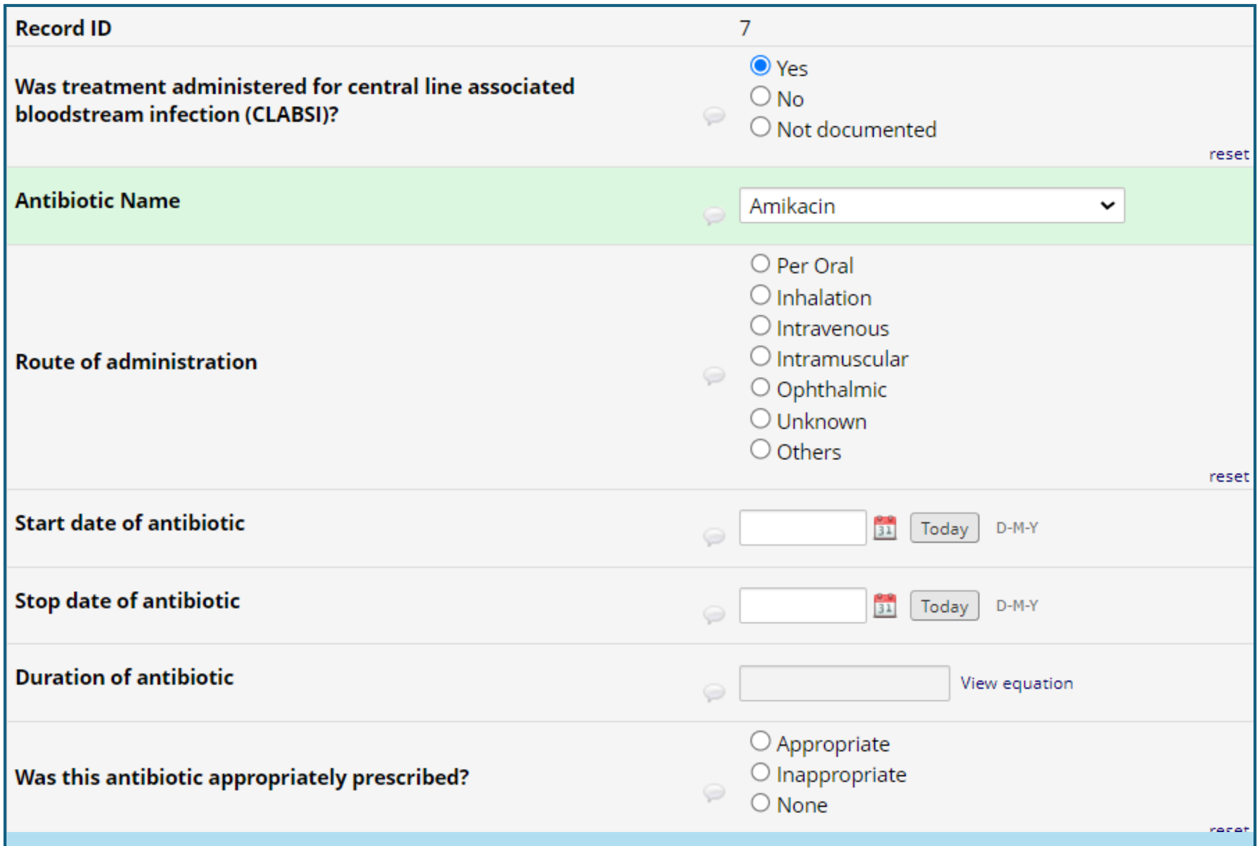

**Record ID**	7
**Was treatment administered for central line associated bloodstream infection (CLABSI)?**	◉ Yes<br>○ No<br>○ Not documented<br>reset
**Antibiotic Name**	Amikacin
**Route of administration**	○ Per Oral<br>○ Inhalation<br>○ Intravenous<br>○ Intramuscular<br>○ Ophthalmic<br>○ Unknown<br>○ Others<br>reset
**Start date of antibiotic**	Today D-M-Y
**Stop date of antibiotic**	Today D-M-Y
**Duration of antibiotic**	View equation
**Was this antibiotic appropriately prescribed?**	○ Appropriate<br>○ Inappropriate<br>○ None<br>reset

No	Data Field	Instruction for data collection
	Antibiotic Name	Required. If patient was given antibiotic, please enter the name of antibiotic.<br>e.g. Amikacin as shown above.<br>If Others is selected, enter the specific medication name in the field.
	Route of administration	Select the one of the provided route for antibiotic administration.<br>PO = per oral (by mouth); IV=intravenous; IH=inhalation; IM=intramuscular; OPH=ophthalmic; UNK=unknown; OTH=others<br>Provide the name of the route if Others is selected
	Start date of antibiotic	Enter the date of antibiotic start using this format: DD/MM/YYYY
	Stop date of antibiotic	Enter the date of culture taken using this format: DD/MM/YYYY
	Duration of antibiotic	The duration of antibiotic will be auto calculated

	Was this antibiotic appropriately prescribed?	Specify if the antibiotic given is appropriately prescribed or inappropriate. If unsure, please kindly ask your specialist or consultant.<br><br>An antibiotic is considered appropriate when it was prescribed from up to 2 days prior to infection onset, and able to treat the organism(s) grown in the microbiology culture taken to determine the infection episode.

Antibiotic Resistance and Previous Exposure

**Antibiotic resistance (colonized or infected)**<br>**If gram positive bacteria: 1 years & gram negative bacteria : 3 months**	○ Yes<br>○ No<br>reset<br>Antibiotic resistance: The ability of bacteria and other microorganisms to resist the effects of an antibiotic to which they were once sensitive.
**Previous antibiotic Exposure for past 3 months**	○ Yes<br>○ No<br>○ Not known<br>reset

No	Data Field	Instruction for data collection
	Antibiotic Resistance	Antibiotic resistance refers to colonized or infected gram (+ve) bacteria is 1 year and gram (-ve) bacteria is 3 months except for CRE & VRE is 1 years. Specify Yes or No.<br><br>Antibiotic resistance: The ability of bacteria and other microorganisms to resist the effects of antibiotic to which they were once sensitive. Antimicrobial resistance happens when germs like bacteria and fungi develop the ability to defeat the drugs designed to kill them. That means the germs are not killed and continue to grow. Gram-positive infections include methicillin-resistant Staphylococcus aureus (MRSA), strep infections, and toxic shock. Gram-negative infections include salmonella, pneumonia, urinary tract infections, and gonorrhea. Gram-negative bacteria are resistant to multiple drugs and are increasingly resistant to most available antibiotics. These bacteria have built-in abilities to find new ways to be resistant

		and can pass along genetic materials that allow other bacteria to become drug-resistant as well. Gram-negative infections include those caused by Klebsiella, Acinetobacter, Pseudomonas aeruginosa, and E. coli., as well as many other less common bacteria.

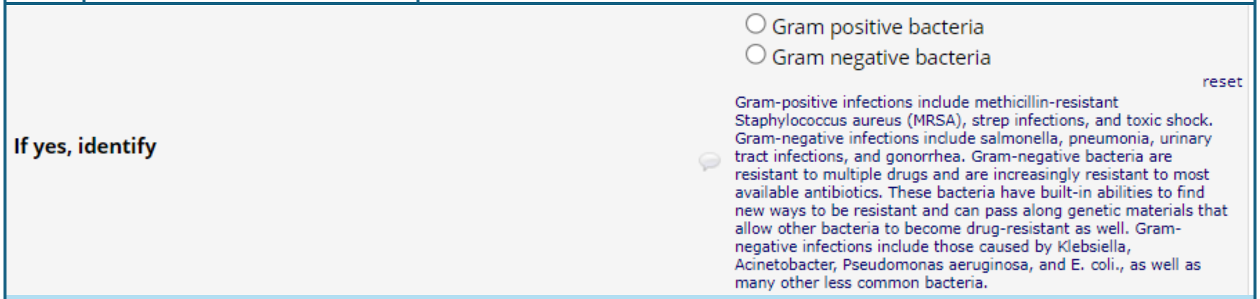

No	Data Field	Instruction for data collection
	If yes, identify	Select either Gram positive bacteria or Gram negative bacteria.

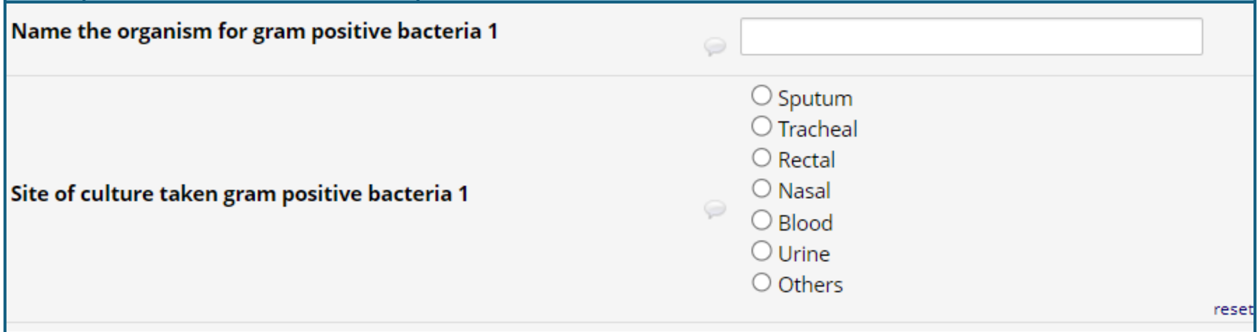

No	Data Field	Instruction for data collection
	Name of the organisms for gram positive bacteria.	Specify the name of organism. Up to three organisms of gram negative may be reported.
	Site of culture taken for gram positive bacteria	Specify from the drop-down list:<br>1. Sputum<br>2. Tracheal aspirate<br>3. Rectal<br>4. Nasal<br>5. Blood<br>6. Urine<br>7. Other, please specify the name of other site of culture

		taken………………………………
	Name of the organisms for gram negative bacteria.	Specify the name of organism. Up to three organisms of gram negative may be reported.
	Site of culture taken for gram negative bacteria	Specify from the drop-down list:<br><br>1. Sputum<br>2. Tracheal aspirate<br>3. Rectal<br>4. Nasal<br>5. Blood<br>6. Urine<br>7. Other, please specify the name of other site of culture taken………………………………

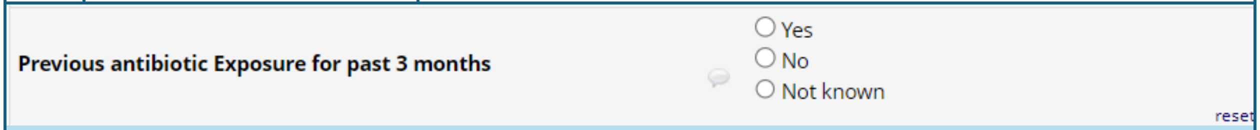

No	Data Field	Instruction for data collection
	Previous antibiotic exposure for past 3 months	Specify Yes, No or Not known if patient were given antibiotic during previous infection/admission.
	Please tick the previous antibiotics exposure past 3 months (could be more than one)	Specify from the drop-down list:<br><br>1. Conventional penicillin<br>2. Cloxacillin<br>3. Ampicillin / Amoxycillin<br>4. Augmentin / Unasyn<br>5. Tazocin (Pip+tazobactam)<br>6. Aminoglycosides (gentamycin, amikacin, streptomycin)<br>7, Vancomycin<br>7. 1st gen ceph (cefazolin, cephalexin)

		8. 2nd gen ceph (cefuroxime, zinnat)<br>9. 3rd gen ceph (ceftriaxone, rocephine, ceftazidime, fortum, defaparazone, cefobid)<br>10. 4th gen ceph (cefipime, maxipime)<br>11. 5th gen ceph (ceftaroline, zinforo)<br>12. Clindamycin<br>13. Metronidazole<br>14. Colistin<br>15. Polymyxin B<br>16. Imipenem / Meropenem<br>17. Ertapenem<br>18. Macrolides (azithromycin, clarithromycin, erythromycin, EES)<br>19. FloroQ (Ciprofloxacin, ciprobay, levofloxacin, moxifloxacin, avelox)<br>20. Daptomycin<br>21. Linezolid<br>22. Nitrofurantoin<br>23. Bactrim<br>24. No known<br>25. Others, please state ………………………………

F06: Outcome

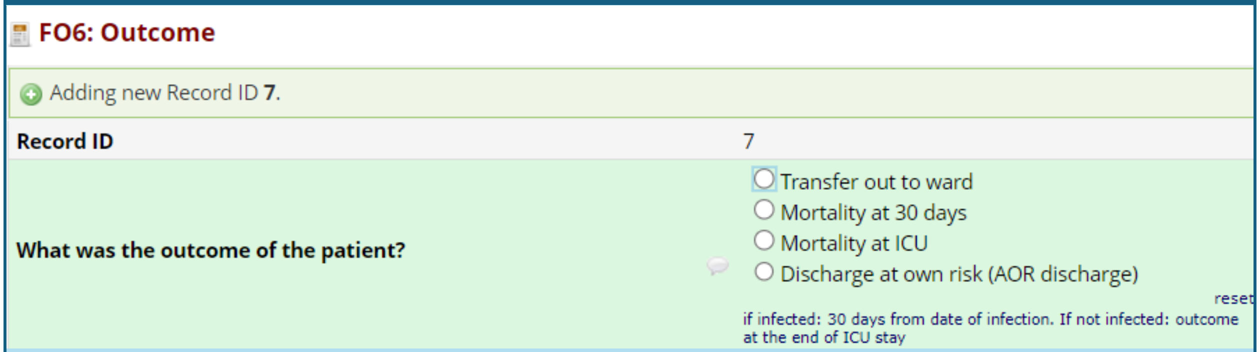
F06: Outcome

Adding new Record ID 7.

Record ID	7
What was the outcome of the patient?	○ Transfer out to ward<br>○ Mortality at 30 days<br>○ Mortality at ICU<br>○ Discharge at own risk (AOR discharge)<br>reset<br>if infected: 30 days from date of infection. If not infected: outcome at the end of ICU stay

No	Data Field	Instruction for data collection
	What was the outcome for the	Specify if the patient is discharged from ICU or

	patient?	hospitalized.<br><br>*If infected: 30 days from date of infection<br>*If not infected: outcome at end of ICU stay
	If transfer out of ward	Specify Alive or Dead or Unable to contact. If dead, please state date using this format: DD/MM/YYYY
	Mortality at ICU	Enter date of mortality above 30 days ICU admission.
