# Supplementary material for: Incidence, Compliance, and Risk Factor Associated with Central Line-Associated Bloodstream Infection (CLABSI) in Intensive Care Unit (ICU) Patients: A Multicenter Study in an Upper Middle-Income Country
Source: Antibiotics (Basel). 2025 Mar 7;14(3):271. doi: 10.3390/antibiotics14030271 (PMC11939773; doi:10.3390/antibiotics14030271)
Supplement: Supplementary file 1 [file antibiotics-14-00271-s001.zip › antibiotics-3477605-supplementary-Table S1.pdf]

Supplementary File 2

Table S1: Compliance of Individual CVC Care Bundle (CCB) and Overall compliance (N=997)

Month	Total catheter	Hand hygiene		Maximal Barrier Precaution		Chlorohexidine Antisepsis		Daily Review		Overall CVC compliance	
		n	%	n	%	n	%	n	%	n	%
**Oct-2022**	265	179	67.55	179	67.55	172	64.91	188	70.94	**172**	**64.91**
**Nov-2022**	252	179	71.03	178	70.63	172	68.25	215	85.32	**172**	**68.25**
**Dec-2022**	246	173	70.33	170	69.11	162	65.85	178	72.36	**162**	**65.85**
**Jan-2023**	234	162	69.23	161	68.80	161	68.80	188	80.34	**147**	**62.82**
**Total**	**997**	**693**	**69.51**	**688**	**69.01**	**667**	**66.90**	**769**	**77.13**	**653**	**65.50**
